# Nuclear Egress Complexes of HCMV and Other Herpesviruses: Solving the Puzzle of Sequence Coevolution, Conserved Structures and Subfamily-Spanning Binding Properties

**DOI:** 10.3390/v12060683

**Published:** 2020-06-24

**Authors:** Manfred Marschall, Sigrun Häge, Marcus Conrad, Sewar Alkhashrom, Jintawee Kicuntod, Johannes Schweininger, Mark Kriegel, Josephine Lösing, Julia Tillmanns, Frank Neipel, Jutta Eichler, Yves A. Muller, Heinrich Sticht

**Affiliations:** 1 Institute for Clinical and Molecular Virology, Friedrich-Alexander University of Erlangen-Nürnberg, Medical Center, 91054 Erlangen, Germany; sigrun.haege@fau.de (S.H.); jintawee.kicuntod@extern.uk-erlangen.de (J.K.); josi.loesing@fau.de (J.L.); jul.tillmanns@fau.de (J.T.); frank.neipel@fau.de (F.N.); 2 Division of Bioinformatics, Institute of Biochemistry, Friedrich-Alexander University of Erlangen-Nürnberg, 91054 Erlangen, Germany; mar.conrad@fau.de (M.C.); heinrich.sticht@fau.de (H.S.); 3 Division of Medicinal Chemistry, Department of Chemistry and Pharmacy, Friedrich-Alexander University of Erlangen-Nürnberg, 91058 Erlangen, Germany; sewar.alkhashrom@fau.de (S.A.); jutta.eichler@fau.de (J.E.); 4 Division of Biotechnology, Department of Biology, Friedrich-Alexander University of Erlangen-Nürnberg (FAU), 91052 Erlangen, Germany; johannes.schweininger@fau.de (J.S.); mark.kriegel@fau.de (M.K.); yves.muller@fau.de (Y.A.M.)

**Keywords:** human cytomegalovirus (HCMV), nuclear egress complex (NEC), core NEC crystal structures, α-, β-, γ-herpesviral NECs, sequence coevolution, highly conserved structures, subfamily-specific binding properties, regulators of viral replication and pathogenicity, novel NEC-directed antivirals

## Abstract

Herpesviruses uniquely express two essential nuclear egress-regulating proteins forming a heterodimeric nuclear egress complex (core NEC). These core NECs serve as hexameric lattice-structured platforms for capsid docking and recruit viral and cellular NEC-associated factors that jointly exert nuclear lamina as well as membrane-rearranging functions (multicomponent NEC). The regulation of nuclear egress has been profoundly analyzed for murine and human cytomegaloviruses (CMVs) on a mechanistic basis, followed by the description of core NEC crystal structures, first for HCMV, then HSV-1, PRV and EBV. Interestingly, the highly conserved structural domains of these proteins stand in contrast to a very limited sequence conservation of the key amino acids within core NEC-binding interfaces. Even more surprising, although a high functional consistency was found when regarding the basic role of NECs in nuclear egress, a clear specification was identified regarding the limited, subfamily-spanning binding properties of core NEC pairs and NEC multicomponent proteins. This review summarizes the evolving picture of the relationship between sequence coevolution, structural conservation and properties of NEC interaction, comparing HCMV to α-, β- and γ-herpesviruses. Since NECs represent substantially important elements of herpesviral replication that are considered as drug-accessible targets, their putative translational use for antiviral strategies is discussed.

## 1. Introduction

Human cytomegalovirus (HCMV) is a major human pathogenic β-herpesvirus with ubiquitous, worldwide distribution causing life-long latent infection in the host. Seroprevalence ranges from approx. 50% to more than 95% in individual regional populations. Primary infection with HCMV frequently remains asymptomatic in the immunocompetent host, associated with productive virus replication and shedding in body fluids before viral latency is established. HCMV reactivation and reinfection may occasionally occur, either asymptomatic or accompanied by mild febrile illness [1,2]. In the immunonaïve unborn or infant, however, HCMV may cause severe or even life-threatening courses of infection. Specifically, congenital HCMV infection (cCMV) acquired during pregnancy represents a serious medical problem, frequently leading to severe cytomegalovirus inclusion disease and developmental defects such as sensorineural hearing loss (SNHL), mental retardation or microcephaly. To date, HCMV represents the most frequent cause of pathogen-derived developmental defects during pregnancy [2,3]. Moreover, in immunosuppressed individuals, e.g., transplant recipients, tumor and AIDS patients, HCMV infection can likewise lead to severe symptoms and HCMV itself may further weaken immune responses [4]. Thus, HCMV disease manifestations can range from self-limiting febrile periods to fatal end-organ disease. So far, no vaccine has been approved for the prevention of HCMV infections and although anti-HCMV drugs are available, problems of unwarranted side-effects or drug-induced viral resistance mutations interfere with the success of therapy and the mechanistic repertoire of approved drugs is still limited [5,6,7]. This underlines the need for novel anticytomegaloviral/antiherpesviral strategies and accessible target proteins.

The herpesviral nuclear egress complex (NEC) is a fascinating example in how a viral heterodimeric element (core NEC) can represent a scaffold, recruiting an entity of cellular and viral NEC-associated proteins (multicomponent NEC), to hijack host-specific functions of protein transport and intracellular trafficking [8]. The three main steps in regulating herpesviral nuclear egress are (i) the formation of a multicomponent NEC, (ii) the phosphorylation-induced reorganization of the nuclear lamina and (iii) the NEC docking and nuclear membrane budding of viral capsids. This mechanism is balanced by two virus-encoded core NEC proteins, i.e., pUL50 and pUL53 of HCMV or herpesviral homologs. The recruitment of cellular and viral proteins like protein kinases (pUL97, PKC, CDK1, possibly others [8,9]) by the core NEC leads to the formation of the multicomponent NEC. Site-specific phosphorylation of the nuclear lamins by NEC-associated kinases results in massive rearrangement of the nuclear envelope and particularly the formation of lamina-depleted areas (LDAs), the sites where viral nuclear capsids gain access to the nuclear envelope [10,11,12]. Additional events of nuclear envelope reorganization, including the formation of a hexameric NEC coat and patch-like lattice within the LDAs, apparently serving as a platform for capsid docking, allows the budding of the intranuclear HCMV/herpesviral capsids into the perinuclear space [13]. Hitherto, mainly the regulation of the nuclear egress of individual herpesviruses has been mechanistically investigated and a number of NEC-associated effector proteins have been identified [9,14,15]. Additionally, structural investigations revealed wide-ranging similarities between α-, β- and γ-herpesviral NECs [16,17,18,19,20,21,22,23,24]. The 3D structures of four different herpesviral core NECs have been crystallographically determined by independent groups [16,20,22,23,24]. The multiple ways of interaction between viral NEC proteins and host cell factors, including their functional impact, is a key issue in understanding this essential process in herpesviral replication, also representing a rate-limiting step in virus-induced pathogenesis.

Currently, a focus of many researchers is directed at the study of multiprotein complexes assembled between virus and host proteins, so that investigations of the HCMV-specific NEC appear highly relevant in this regard (for methods, see Supplementary Materials [25,26,27,28,29]). Novel information is accumulating for the biochemical and functional properties of NECs, raising the option to define mechanistic ways of a pharmacological interference with herpesviral nuclear egress, which may provide novel targeting strategies for NEC-directed small molecules.

## 2. The NEC, a Unique Nuclear Egress-Regulating Multiprotein Complex of Herpesviruses

**Figure 1:**
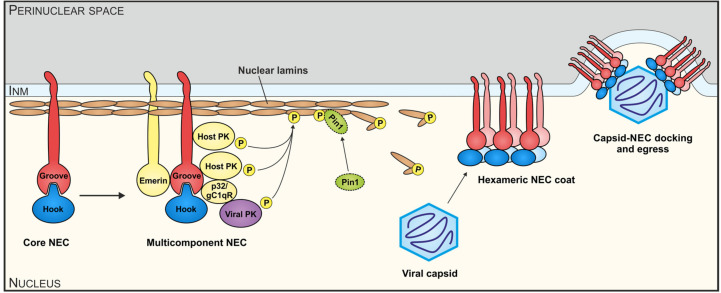
Schematic illustration of functional aspects of the herpesviral nuclear egress complex (NEC) protein interactions and the multiple regulatory aspects of the nuclear egress-specific sequence of events. In essence, the initially formed core NEC and the assembled multicomponent NEC provide the basis for nuclear lamina as well as membrane-rearranging functions and the formation of a hexameric NEC coat, which then serves as a platform for capsid docking. Notably, viral and cellular protein kinases (PKs, i.e., pUL97, PKC, CDK1 and other enzymatic regulators, like the prolyl cis/trans isomerase Pin1), have been identified in the case of human cytomegalovirus (HCMV) (reviewed in Marschall et al. [8]). They represent important active components by site-specific phosphorylation and structural modulation of nuclear lamins A/C and also NEC components. As a result of this concerted activity of the multifunctional NEC, viral nuclear capsids are guided to lamina-depleted areas, where they undergo budding and primary envelopment at the inner nuclear membrane (INM). This scheme represents a refined version of a general herpesviral NEC model as analogously published before for the specific case of HCMV [25].

Herpesviral intracellular replication faces the nuclear envelope as a physical barrier that separates the nucleus from the cytoplasm. Since herpesviral genomic replication starts in the host cell nucleus, where preformed capsids are packaged and exported to the cytoplasm for further virion maturation, the transition of capsids through the nuclear envelope represents a rate-limiting step termed nuclear egress that combines several regulatory processes [8,30,31,32,33,34,35,36,37,38,39,40,41]. As a central aspect of the regulated nuclear egress, the nuclear envelope is reorganized at specific sites with a profound importance of phosphorylation-triggered distortion of the nuclear lamina [42,43,44]. To orchestrate this sequence of highly coordinated processes, in particular based on a number of protein–protein interactions, a defined multiregulatory NEC is assembled, comprising both viral and cellular components. A core element of the NEC is comprised by two essential viral proteins that heterodimerize and thus form an assembly scaffold at the inner side of the nuclear lamina. The NEC regulates important steps of herpesviral nuclear replication, i.e., recruitment of NEC-associated effector proteins, reorganization of the nuclear lamina and membranes, and the docking of nuclear capsids (Figure 1). Consequently, the multiple functions assembled by the NEC give rise to the stepwise transition of viral capsids through the nuclear membranes (envelopment–deenvelopment–reenvelopment), cytoplasmic morphogenesis, tegumentation at cytoplasmic viral assembly compartments, and the release of mature virions [8,45].

## 3. Recognizing the Importance of a Finely Regulated Process of Nucleocytoplasmic Egress for the Efficiency of Herpesviral Replication

The principle of herpesviral nuclear egress has just been recognized barely twenty years ago, when the barrier function of the nuclear lamina was investigated in greater detail. Then it became evident that the rate of nuclear membrane budding of herpesviral nucleocapsids strictly depends on the preceding capsid transition through the nuclear lamina, which requires essential, non-membrane-directed measures of reorganization. Additionally, the steps succeeding nuclear envelope transition, namely the cytoplasmic virion morphogenesis, based on a complex mode of envelopment–deenvelopment–reenvelopment has been largely reconsidered [31,32,33,34,41]. Specifically, the regulation of nuclear egress, as based on nuclear lamina reorganization, has first been mechanistically analyzed for cytomegaloviruses [12,46,47,48]. In this context, the HCMV-encoded protein kinase pUL97 was identified as the first herpesviral kinase with lamin-phosphorylating activity [49]. Additionally, central importance has also been pointed out by recent investigations of the recruitment of lamin-phosphorylating viral and cellular kinases as well as further lamin-modifying proteins, such as prolyl cis/trans isomerase Pin1 [10]. For achieving this mode of recruitment, the heterodimeric core NEC proteins form a rim-like association at the inner side of the nuclear envelope (e.g., pUL34-pUL31 for HSV-1, pUL50-pUL53 for HCMV, and BFRF1-BFLF2 for EBV) in order to provide a scaffold for the assembly of various NEC-associated enzymes and regulatory factors. Consequently, the multiple functions assembled into the multicomponent NEC give rise to the stepwise transition of viral capsids through the nuclear membranes, to subsequent cytoplasmic morphogenesis including capsid tegumentation at cytoplasmic viral assembly compartments and the release of mature virions [45]. 

As far as the reversible disassembly of the nuclear lamina is concerned, similar processes were described for cellular processes including mitosis and nuclear export of large messenger ribonucleoprotein complexes [50,51]. Specifically, during HCMV replication, the nuclear lamina is locally distorted, mainly on the basis of a disassembly of lamins A/C induced by the site-specific phosphorylation at Ser22 (in part also at Ser392). At present, the question of which protein kinases contribute to the overall phosphorylation of the nuclear lamina and the NEC itself in HCMV-infected cells remains unanswered, although the viral protein kinase pUL97 definitely plays a predominant role. In general terms, a number of lamin-phosphorylating protein kinase activities have been described, including cyclin-dependent kinase (CDK) 1, protein kinase C (PKC), protein kinase B, and CDK-like enzymes, including herpesviral protein kinases. Thus, the regulation of nuclear egress appears to be a result of the concerted action between various viral and cellular NEC-associated kinases and other proteins. Specifically, the herpesviral core NEC, e.g., HCMV pUL50-pUL53, recruits a number of homologous herpesviral UL-type protein kinases and nonhomologous viral proteins as well as cellular proteins. According to our current knowledge, NEC-associated cellular proteins are composed of typical nuclear lamina/envelope proteins, mutiligand-binding bridging factors such as p32/gC1qR, nuclear transport factors, protein kinases, a prolyl cis/trans isomerase and other regulatory factors (reviewed in [8,44]). An unexpected finding was that there are both, identical and dissimilar features concerning the overall composition of the multimeric NECs between α-, β-, and γ-herpesviruses, considering the fact that the basic features of NEC functionality and nuclear envelope remodeling are very similar. 

## 4. Definition of Biochemical and Functional Components of the Cytomegalovirus-Specific NEC

As far as nuclear egress of HCMV is concerned, a nuclear rim corecruitment of the NEC core proteins pUL50 and pUL53 has been described in detail [21,26,52]. Distinct mechanisms of INM-targeting were proposed for pUL50 and pUL53, as studied on the basis of infection kinetics using confocal laser-scanning microscopy [28]. HCMV pUL53 is initially translocated into the nucleus via the classical nuclear import pathway, while pUL50 seems to be transported to the INM by membrane-bound translocation from its insertion site at the endoplasmic reticulum along nuclear pores. Interestingly, recent analysis of HSV-1 core NEC homologs confirmed that pUL31 precedes pUL34 in nuclear import and that proteins do not associate yet in the cytoplasm [53]. Instead, the HSV-1 proteins pUL34 and pUL31 form a core NEC by heterodimerization at the nuclear rim, before they recruit further viral and cellular proteins [11,54]. As determined by proteomic analyses, the main constituents of the cytomegalovirus-specific multi-component NEC are the virus-encoded protein kinase, p32/gC1qR, emerin, PKC, and additional proteins [27,55,56]. Among these identified NEC constituents, pUL97 is of particular importance, because its kinase activity is primarily responsible for nuclear lamina disassembly during the late phase of HCMV replication (reviewed in [44]). It is primarily pUL97 that is recruited to the nuclear lamina for site-specific phosphorylation and consequent disruption of the lamina, as studies from independent investigators clearly demonstrated [49,57]. A more detailed analysis of protein–protein interactions demonstrated that the NEC association of pUL97 is mostly mediated in an indirect way through p32/gC1qR binding, thus bridging pUL97 to the pUL50-pUL53 core NEC [58]. In addition, cellular protein kinases were found to be NEC-associated, in particular, PKC and CDK1, as identified by pUL50-specific coimmunoprecipitation [26,54,58]. However, the activity of cellular kinases PKC and CDK1 is most probably not directly targeted to nuclear lamins, because pUL97 was shown to be primarily responsible for the site-specific lamin phosphorylation [27,57]. Instead, these recruited protein kinases may be required for proper NEC formation, including the so far poorly understood events of regulatory phosphorylation of NEC-associated proteins [26,59].

## 5. Comparison of Primary Sequences and Structural Properties between the Core NECs of HCMV and other α-, β- and γ-Herpesviruses

**Figure 2:**
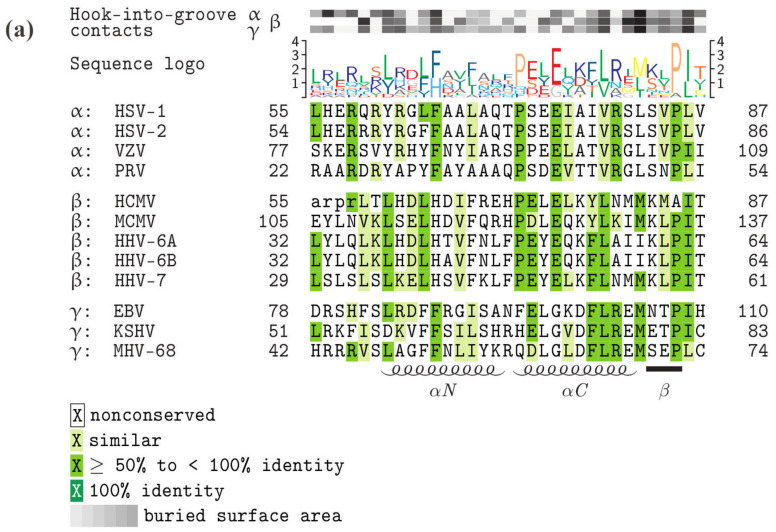

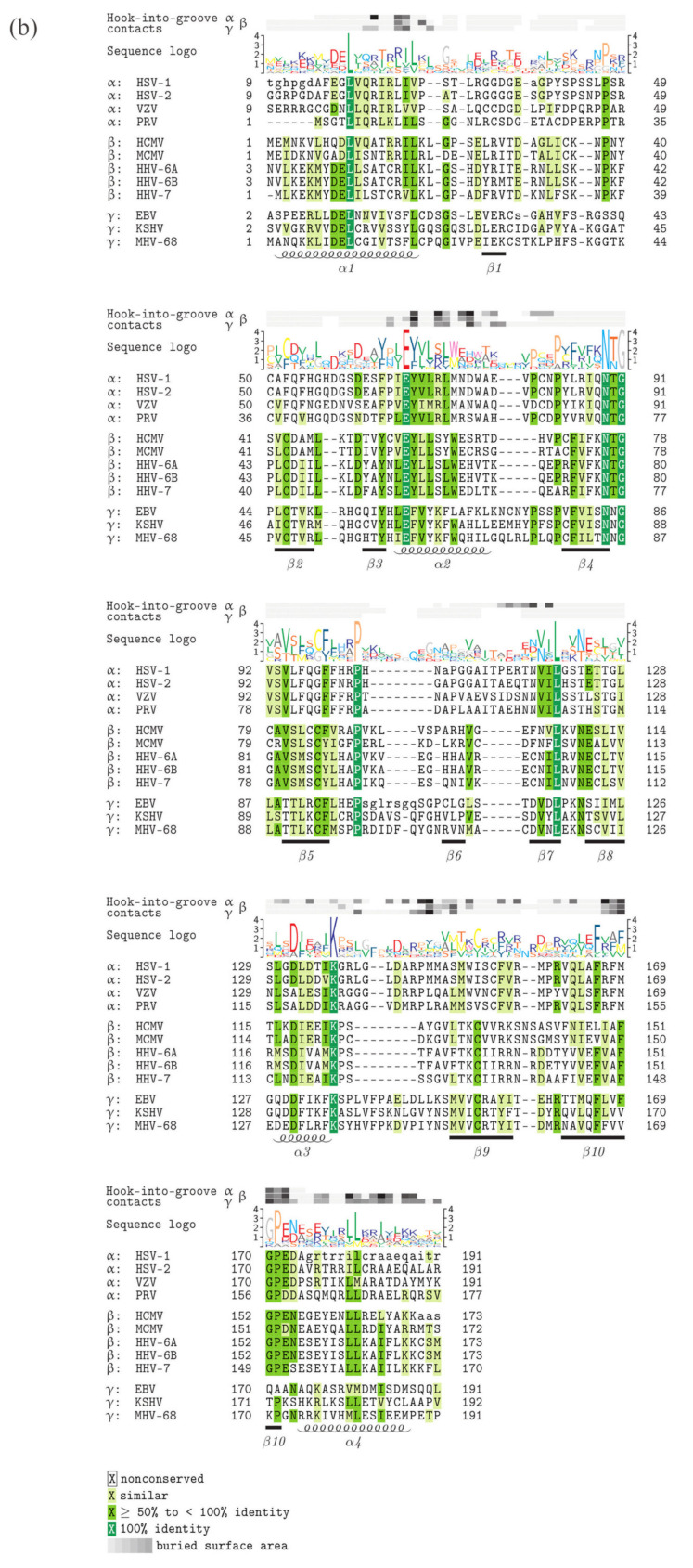
Core NEC protein sequence alignments, highlighting the functionally important hook and groove domains, conserved amino acids and amino acids representing contact interfaces. (**a**) Homologs of HCMV pUL53 (hook proteins), (**b**) homologs of HCMV pUL50 (groove proteins). Increasing levels of sequence conservation are indicated by darker shades of green in the alignment and by higher letters in the sequence logo above the alignment. Lower-case letters mark those residues that were not resolved in the crystal structures of the respective proteins. The buried surface area at hook-into-groove contact positions in α-, β-, and γ-herpesviruses is indicated by gray squares. Darker shades of gray indicate a larger buried surface. The elements of secondary structure are depicted schematically below the alignment. GenbankTM accession numbers: HSV-1, P10218 and P10215; HSV-2, P89457 and P89454; VZV, P09280 and P09283; PRV (SuHV-1), T2FKZ7 and G3G955; HCMV, P16791 and P16794; MCMV (MuHV-1), D3XDN8 and D3XDP1; HHV-6A, P52465 and P28865; HHV-6B, Q9QJ35 and Q9WT27; HHV-7, P52466 and P52361; EBV, P03185 and P0CK47; KSHV (HHV-8), F5HA27 and F5H982; MHV-68 (MuHV-4), O41968 and O41970.

As far as the conservation of primary sequences of the core NEC proteins is concerned, a stepwise graduation of levels of conservation was recently reported. Members of the subfamilies α-, β- and γ-*Herpesvirinae* showed marked differences in sequence characteristics. While strains of HCMV showed highly conserved sequences for pUL50 and pUL53 (98.5%–99.5% and 98.4%–100%, respectively), the comparison between HCMVs and primate human cytomegaloviruses (CMVs) or rodent CMVs showed substantially decreasing conservation levels [25]. Even the comparison with human roseoloviruses (HHV-6A, HHV-6B and HHV-7) underlined the poor core NEC amino acid identities with human CMVs (≤ 25% for pUL50 and < 32% for pUL53). The alignments of pUL53 (Figure 2a) and pUL50 homologs (Figure 2b) illustrate the sequence conservation of α-, β- and γ-herpesviral core NEC proteins. The primary sequences defining the globular domain, in particular the associated structural elements of the hook or groove elements, are depicted in their similarity to each other, as illustrated by the sequence logo shown above the alignments. A basic and unexpected finding was that within these elements the number of identical amino acids is rather small (none in pUL53-hook, seven in pUL50-groove homologs). Likewise, the number of highly similar residues is limited, although it shows there is some clustering in their sequence distribution. This clustering of similar residues shows some collinearity with the hook-into-groove contact residues and interface stretches (see grey labeling of buried surface area, Figure 2) in several sequence portions, but are independent from each other in some other portions. Importantly, the main contact residues are frequently not those residues which exhibit the highest sequence conservation in the alignment. Thus, it is fascinating to address the question to which extent the core NEC sequence–structure relationship predefines NEC functionality. The hallmarks described above strongly suggest that the functional consistency of core NEC proteins may be primarily based on common structural features, but is obviously not mirrored by the level of sequence conservation.

**Figure 3:**
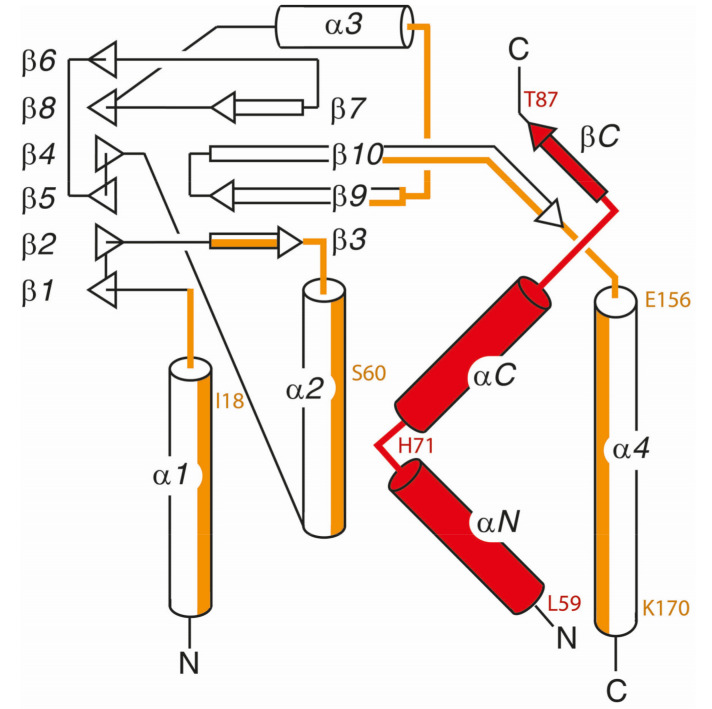
Conserved overall topology and secondary structure content displayed by all herpesviral core NEC structures. The segments of the pUL50 homologous groove proteins that interact with the pUL53 homologous hook segment (in red) are marked in orange. The color code depicts the main regions of hook-into-groove contacts, as similarly marked for the respective contact residues in Figure 4. To improve viewing, the location of some amino acids from HCMV pUL50 and pUL53 is indicated explicitly (see also Figure 4c).

**Figure 4:**
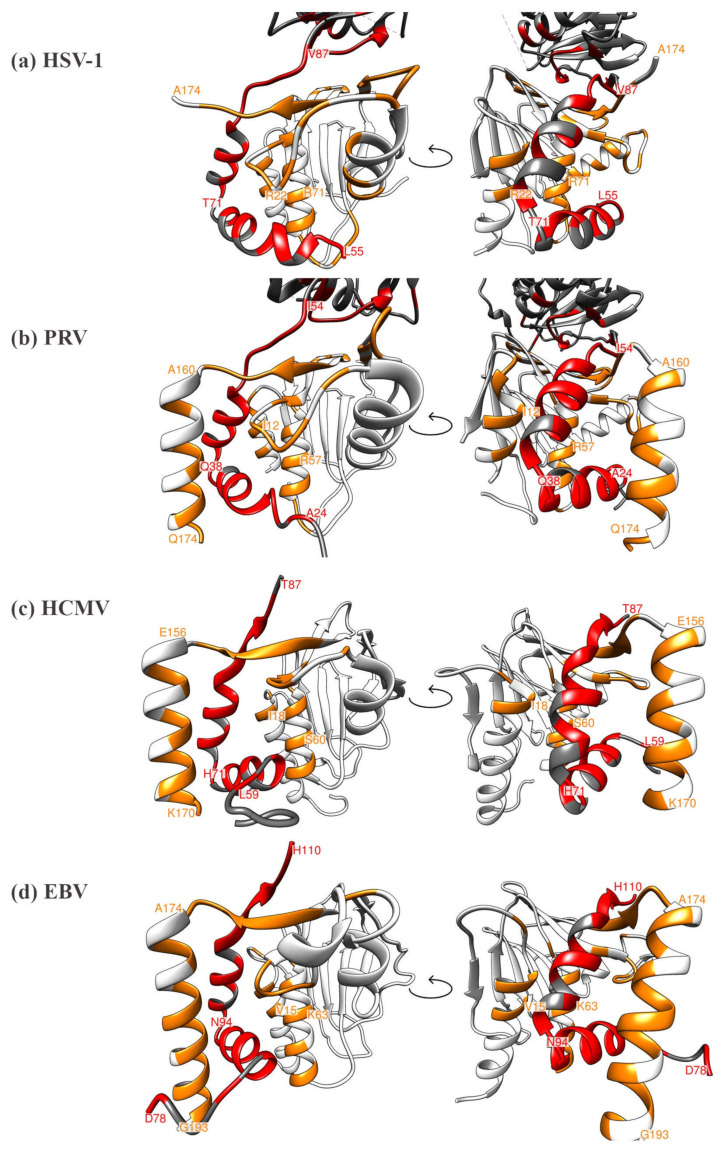
Comparison of the currently available four herpesviral 3D core NEC crystal structures, highlighting the heterodimeric contact interfaces, each in two different viewing angles (left and right rotated by 90 degrees). (**a**) HSV-1 core NEC, (**b**) PRV core NEC, (**c**) HCMV core NEC, (**d**) EBV core NEC. Interacting residues within the hook or groove are colored in red and orange, respectively. Distinct amino acids marking the start or end positions of structural elements are labeled as orientation points.

Regarding the currently available data on core NEC structures, four 3D crystal structures have been published, i.e., those of HCMV, HSV-1, PRV and EBV [16,18,20,22,23,24]. Many structural properties of core NEC proteins were found conserved and qualitatively mostly consistent. All structures display a highly similar element of hook-into-groove interaction. In all cases, the groove proteins are formed by identical secondary structure elements, namely four helical segments and an almost all-antiparallel β-sheet sandwich formed by a 6-stranded and 4-stranded β-sheet (Figure 3). The same also holds true for the hook segment, which in all cases consists of two consecutive α-helices that are followed by a short β-strand. Structural differences between the various complexes are limited to differences in the lengths of the β-strands and α-helices as well as to differences in the lengths and conformations of the loop segments that interconnect the secondary structure elements. The conserved overall topology also holds true for the topological arrangement of the individual interaction patches that mediate the binding of the hook protein to the groove protein. However, it was rather unexpected that both the specific amino acids of the hook-into-groove contact interfaces as well as the invariant fully conserved amino acids are not identical between the compared herpesviruses (Figure 4; Figure S1). When considering the details of structural features, such as helical portions, lengths of beta sheets, unstructured stretches, localization of essential amino acids and others, the entity of structural properties mostly shows high similarity with only some examples of minor differences [22].

Interestingly, although this principle of hook-into-groove interaction is found in an almost identical structural fashion in all core NECs analyzed so far, the individual identity and positioning of amino acids can underlie variability and thus, no fully consistent, simple correlation can be deduced from the comparison between structural elements and the primary sequence alignment. At present, it is not clear, whether the overall consistency of these herpesviral heterodimeric protein pairs in fulfilling the core NEC function also allows some degree of differences in terms of binding NEC-associated proteins, bridging components and nuclear capsids in a virus-/subfamily-specific manner. Additionally, more information will be required to answer the question of whether such individual, differential protein-binding properties may reflect the pronounced sequence differences or some specified structural differences or both.

## 6. Comparative Experimental Assessment of Herpesviral Core NEC Protein Interactions 

**Figure 5:**
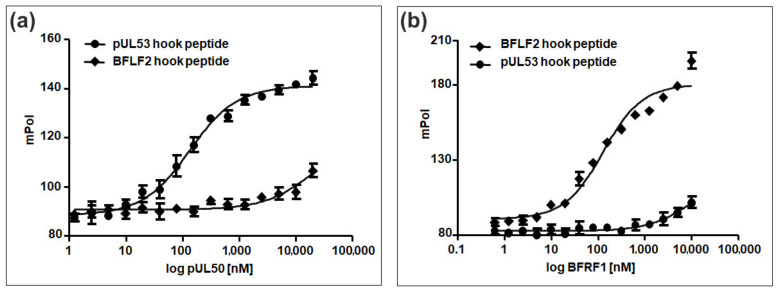
HCMV-/EBV-specific core NEC interaction properties measured in a hook-into-groove binding assay. (**a**) Binding to HCMV pUL50: the pUL53 hook peptide (see Table S1 for peptide sequences), but not the BFLF2 hook peptide, is able to interact with the HCMV groove protein pUL50. (**b**) Binding to EBV BRFR1: likewise, the BFLF2 hook peptide, but not the pUL53 hook peptide, is able to interact with the EBV groove protein BFRF1. All interactions were measured by the fluorescence polarization assay using fluorescein-labeled synthetic hook peptides in conjunction with recombinant groove proteins produced in *E. coli*.

Based on the established hook-into-groove interactions of herpesviral core NEC proteins, we could recently show that synthetic peptides presenting the hook regions of HCMV pUL53 and EBV BFLF2 are able to interact with their cognate groove proteins, i.e., pUL50 and BFRF1, respectively. However, the affinity of the BFLF2 hook peptide for BFRF1 appeared to be considerably lower than that of the pUL53 hook peptide for pUL50 [21]. We have now extended the BFLF2 hook peptide by two amino acids at the N-terminus (D78 and R79), as well as at the C-terminus (I109 and H110). This extended peptide has now shown to interact with BFRF1 with essentially the same affinity (K_D_ = 117 nM) as that for the interaction of the pUL53 peptide with pUL50 (K_D_ = 120 nM) (Figure 5), indicating a strong contribution of the flanking amino acids, which were missing in the previously used BFLF2 hook peptide, to the interaction with BFRF1. Furthermore, essentially no nonautologous binding of the pUL53 hook peptide to BFRF1, and the BFLF2 hook peptide to pUL50, respectively, could be detected, reconfirming the previously postulated strong selectivity of the hook-into-groove interaction between HCMV and EBV, despite the strong structural similarities between the two core NEC complexes. 

In addition, we performed a detailed analysis of the HCMV core NEC complex in order to map the contribution of individual amino acids of the pUL53 hook peptide to the interaction with the pUL50 groove protein. Using experimental and in silico alanine-scanning analyses of the pUL53 hook peptide, we have previously identified a range of individual positions that are most crucial for the interaction of the peptide with pUL50, in particular L64, F68, L74, E75, Y78, L79 and M82, whose replacement with alanine resulted in a dramatic loss of ability to inhibit the pUL50–pUL53 interaction [22]. In a complementary study, we have now experimentally analyzed a D-amino acid scan of the pUL53 hook peptide, in which each amino acid was individually replaced by its respective D-stereoisomer. These peptides enable the evaluation of the importance of side chain orientation of amino acid to the interaction with pUL50. As for the alanine scan peptides, the ability of the D-amino acid scan peptides to inhibit the pUL50–pUL53 interaction (expressed as IC_50_ values) was compared to that of the wild-type peptide. Interestingly, most of the hot spot residues found in the alanine scan, i.e., L64, L74, E75, Y78, L79 and M82 were also identified using the D-amino acid scan analysis (Table 1), indicating that not only the presence of the respective side chains is important for the interaction of the peptide with pUL50, but also that these side chains have to be presented in the correct orientation. It should be noted, however, that changing the stereochemistry of individual amino acids may result in a loss of structural integrity of the α-helical hook peptide, which may in turn lead to a loss in binding affinity of the peptide. In addition to these consistencies, we also identified a few differences between the results of the alanine and D-amino acid scans. While the alanine scan revealed the aromatic side chain of F68 to be essential, the orientation of this side chain appears to be less crucial, as replacement of F68 with D-phenylalanine results in only partial decrease in inhibitory activity. On the other hand, exchanging H71 and M84 for their D-stereoisomers had a much stronger effect than their replacement with alanine, indicating that changing the side chain orientation of these two amino acids results in peptide conformations that are incompatible of interaction with pUL50.

Regarding the nuclear colocalization of core NEC pairs, specific imaging methods focusing on the hook-into-groove rim recruitment have been described by several researchers [9,10,12,21,25,43,48,53,55,59,60,61,62,63,64,65,66,67,68,69]. Recently, we analyzed and compared the capacities of various NEC pairs of α-, β- and γ-herpesviruses to interact in the form of heterodimeric complexes, both in the autologous manner (which was strongly detectable for each individual NEC pair of the virus species analyzed) and a nonautologous, cross-viral manner. These experiments were based on the transient coexpression of two core NEC proteins, one hook-type homolog and one groove-type homolog. Interestingly, data strongly suggested that nonautologous interaction is frequently detectable between core NEC proteins of herpesviruses within the same subfamily (e.g., HCMV/MCMV or HSV-1/VZV). To the contrary, the testing of nonautologous interaction for NEC pairs of viruses belonging to different subfamilies was basically negative [24].

This claim has now been reinvestigated by using a broader selection of herpesviral core NECs, i.e., α-herpesviral pUL34/Orf24 and pUL31/Orf27 (encoded by HSV-1 or VZV, respectively), β-herpesviral pUL50/pM50 and pUL53/pM53 (encoded by HCMV or MCMV, respectively), and γ-herpesviral BFRF1/Orf67 and BFLF2/Orf69 (encoded by EBV or KSHV, respectively). Importantly, all autologous interaction pairs derived from one specific virus, without exception, showed strong signals of nuclear rim-directed core NEC recruitment. However, nonautologous, cross-viral NEC combinations again showed negative signals of colocalization. Concerning core NEC proteins derived from herpesviruses belonging to the same subfamily, most of the analyzed nonautologous combinations showed partially or fully positive signals of colocalization, thus expressing a subfamily-spanning pattern of binding properties (Table 2). Some of these nonautologous NEC test settings showed clearer positive signals in one combination of the protein pairs, but lower signals in the reciprocal combination. This may be explained by methodological limitations, such as nonoptimal protein folding under conditions of transient overexpression or similar (Figure S2). Thus, data support the previous assumption about subfamily-spanning binding properties of core NEC proteins. Combined, the current investigation added to the previously published concept, based on reports from our groups and additionally observed by others, stating that the NEC protein interaction property is basically conserved [8,14,37,38,39]. Thus, with few exceptions, the patterns of core NEC protein interactions are either virus species-specific or limited to protein pairs occurring within one herpesviral subfamily.

## 7. Specific Functional Properties of NECs and Egress Processes That Are either Shared or Distinct between Herpesviruses

An efficient mode of nuclear egress of herpesviral capsids is mostly based on the multifunctionality of the NEC. Very central NEC functions are the virus-induced promotion of lamina disassembly, the capsid budding at the inner nuclear membrane (INM), and the recruitment of capsids to sites of nuclear egress (Figure 1). In the case of HCMV, a nuclear rim corecruitment of the NEC core proteins pUL50 and pUL53 has been described in detail and this property is shared by all other core NEC pairs investigated so far ([8,25] with references therein). As an important feature, the NEC-associated HCMV protein kinase pUL97 interacts with p32/gC1qR, which bridges pUL97 to the pUL50–pUL53 core NEC [54,58]. A similar bridging function of p32/gC1qR has been suggested for the NECs of other α-, β- and γ-herpesviruses [49,55,70,71]. In addition to HCMV pUL97, cellular protein kinases were found to be NEC-associated, in particular, PKC and CDK1 [9]. Concerning the lamina-specific regulatory effects of kinases, a site-specific phosphorylation of lamins A/C at Ser22 is known to mediate nuclear lamina disassembly both during herpesviral nuclear egress and cellular mitosis [72,73]. In contrast, differences in the site-specific phosphorylation of lamins A/C at Ser389 have been identified for members of the herpesviral subfamilies [10]. Seeking to understand the far-reaching consequences of the distinct mode of lamin phosphorylation, it has been proposed on the basis of lamin A coil 2B dimeric crystal structures that phosphorylation may interfere with electrostatic interactions [74]. This scenario, however, was challenged by the identification of the phosphorylation-dependent lamin binding of the peptidyl-prolyl cis/trans isomerase Pin1 and the functional validation that strongly supported a mode of Pin1-induced conformational change facilitating lamina disassembly. To illustrate this process, the strategies of pharmacological Pin1 inhibition or genetic Pin1 knockout provided conclusive evidence that Pin1 promotes the disassembly of the nuclear lamina during HCMV replication [10,12]. Very recently, we were able to provide additional evidence for the relevance of Pin1 in HCMV replication, in that data derived from several independent approaches demonstrated an interaction of Pin1 with the viral proteins pUL50, pUL69 and pUL44 [75,76]. Whether the regulatory impact of Pin1, especially its lamin-directed role in nuclear egress, is specific for HCMV, or whether Pin1 activity is likewise a regulatory cofactor for other α-, β- and γ-herpesviruses has still to be clarified.

As another interesting aspect, additional NEC activities were demonstrated in studies on nuclear membrane vesicles. Such vesicles could be induced through a coexpression of viral homologs of pUL50 and pUL53 in the absence of virus infection [62,77]. Intriguingly, Bigalke et al. [17] provided evidence that the purified core NEC of HSV-1 is sufficient for membrane budding in vitro. This was demonstrated by applications of fluorescence-based tests and cryo-electron microscopy using large and giant unilamellar vesicles. Hereby, a model was proposed, in which core NEC heterodimers form hexagonal arrays leading to membrane invagination and scission in a cell-free environment. Further investigations used cell-based systems to demonstrate the formation of perinuclear vesicles, further illustrating the ultrastructural architecture of alphaherpesviral NEC hexagonal arrays [18,24]. A current concept suggests that the core NEC-induced membrane deformation, scission, and budding of membrane vesicles is independent from additional viral or cellular proteins. A third postulated function of the NEC, namely, the recruitment of capsids to sites of nuclear egress at the INM, has been investigated by interaction studies on one or both of the HCMV core NEC proteins pUL53 and pUL50 with nuclear capsids. This point is currently still speculative as to whether it is a main function of pUL53, or herpesviral pUL53 homologs, to have initial contact with capsids, so the scenario still requires more detailed evidence. Initial information came from studies with α-herpesviruses [78,79,80]. The HSV-1 homolog of pUL53 binds a complex of the HSV-1-encoded proteins pUL25 and pUL17 of the heterodimeric capsid vertex-specific complex (CVSC) [79,80] and thereby the pUL53 homologs of herpesviruses might confer the contact to nuclear capsids [53]. Support of this motion was provided by an immune-electron microscopic study on HCMV, demonstrating a strong decoration of nuclear capsids with pUL53, and to some lower extent also with pUL97 [11]. Notably, a uniform distribution of pUL50-pUL53 on capsid surfaces appears rather unlikely, because the core NEC may occur in hexagonal arrangements and these may adopt various distances in their membrane-bound form. Accordingly, it has also to be taken into account that the hexagonal core NEC is embedded in a fluid membrane environment, so that the formation of contiguous core NEC coats and the NEC–capsid interaction might be more flexible in distance than initially expected [8]. Very recently, Draganova et al. [13] provided additional evidence to show that the HSV-1 NEC coat interacts with capsids and how curved coats may be generated to enable budding. The authors stated that during nuclear budding, binding of pUL25, situated at the pentagonal capsid vertices, promotes formation of core NEC pentagons at the INM. Consequently, the incorporation of NEC pentagons at the points of contact with the vertices may also promote assembly of the curved hexagonal core NEC coat around the capsids leading to capsid egress. Thus, also in this aspect of NEC functionality, the common concept of nuclear capsid-NEC docking appears to be shared by herpesviruses. Nevertheless, the possibility of differences between virus species or subfamilies, such as an additional role of host factors and/or docking bridging factors, needs to be answered by future experimentation.

## 8. Future Perspective of the Pharmacological Interference with Herpesviral NEC Functions and Their Exploitation as Putative Antiviral Drug Targets

The current medical opportunities of the prevention and control of HCMV infections span several different, yet still unsatisfying options. Although no HCMV vaccine has been approved to date, antiviral drugs are available and applied with principally promising success. Most of the approved anti-HCMV drugs interfere with the viral DNA polymerase pUL54, as represented by nucleoside/nucleotide analogs, such as the gold standard ganciclovir (GCV), its prodrug valganciclovir (VGCV), cidofovir (CDV) and the pyrophosphate analog foscarnet (FOS). As a limiting issue, however, these drugs frequently cause adverse side effects, such as myelotoxicity, anemia and nephrotoxicity, or show poor bioavailability, which drives the selection of drug resistant virus variants [6,81,82,83,84,85,86,87,88]. Fortunately, in 2017, letermovir (LMV) has been successfully assessed in clinical trials, thus representing the first anti-HCMV drug that targets the viral terminase complex consisting of pUL56, pUL89 and pUL51 core-subunits. To date, LMV is approved for HCMV prophylaxis in hematopoietic stem cell transplantation recipients and furthermore represents a promising candidate for future combination therapies or even options of cCMV control [89,90,91,92]. Current points of clinical limitation, however, are the occurrence of LMV-resistant viral mutants [93] and the present lack of an approved treatment option for infants, so that the need for advancements in the development of new antiviral drugs remains.

During recent years, the HCMV-encoded protein kinase pUL97 has been suggested as another highly interesting drug target, such that kinase inhibitors with pUL97 specificity, derived from independent chemical classes, have been profoundly characterized towards their clinical investigation [7]. Maribavir (MBV) is a benzimidazole riboside, structurally related to the terminase inhibitor LMV. However, unlike LMV, MBV is not directed to the HCMV terminase, but is directed against the protein kinase pUL97 and shows outstanding inhibitory activity with very low levels of side/off-target effects [81]. Specifically, MBV exhibits favorable pharmacokinetic properties, is well tolerated, and holds promise as a new drug for the treatment of HCMV infections [94,95,96]. It should be stressed that although MBV-treated patients failed to meet the clinical endpoint objectives in the first phase III clinical study [97], further phase III trials are currently enrolling patients to compare the efficacy of MBV with GCV (NCT02931539, NCT02927067). Still one limitation might also persist with an expected approval of MBV as a next HCMV-specific therapy. This is based on the fact that the inhibition of pUL97 kinase activity by MBV interferes with the activation of GCV, thus resulting in drug antagonism, which might most probably reduce their antiviral efficacies in a combination therapy [98]. Thus, a broader basis of applicable anti-HCMV drugs, in ideal terms acting with different mechanistic modes of antiviral efficacy, is still required. Moreover, the long-held desire to develop a broad-acting pan-antiherpesviral drug for the treatment of more than one herpesvirus-induced pathogenesis, mainly in immune-impaired risk persons, should be addressed in the near future.

The herpesviral NEC is considered as a potential antiviral target, since it appears attractive in various aspects: (i) the NEC fulfills an essential function in the replication cycle of basically all herpesviruses ([8], with few exceptions [42,99]), (ii) core NEC 3D crystal structures are available for α-, β- and γ-herpesviruses (see above, chapter 3.4), and (iii) first prototypes of in vitro NEC inhibitors have been described on the basis of synthetic peptides [22,100] and this antiviral principle might likewise be followed through the NEC-specific screening of small molecules (reviewed in Marschall et al. [8] with references therein). As far as the latter option is concerned, even the potential to achieve broad-spectrum, pan-antiherpesviral activity might be considered through NEC-directed small molecules. The realization of this goal would be particularly useful in clinically complex situations, such as post-transplant immunosuppression, in which a number of different herpesvirus-associated diseases and medical complications can arise [101].

It is generally believed that promising opportunities might open up, particularly in achieving a breadth in antiviral drug activity, by the exploitation of highly conserved viral functions, such as the herpesviral core NEC. In particular, when focusing on the aim to suppress an emergence of antiviral drug resistance, viral multimeric proteins and protein complexes, such as the NEC, might represent dominant drug targets [102]. For this reason, it will be highly challenging to define those targeting points that allow for the positioning of NEC-based pan-antiherpesviral inhibitory small molecules. In particular, steric inhibitors of NEC subunit assembly, either blocking the interaction of core NEC or multimeric NEC-associated factors, may have great potential for exerting a strong and possibly multi-objective blocking activity with putative potential for antiherpesviral pharmacological development [8,30,40,41,101]. Likewise alternative mechanisms of NEC inhibition could be similarly effective, such as an inhibitory targeting of NEC phosphorylation. Considering the fact that most, if not all, of the herpesviral core NEC and NEC-associated proteins undergo phosphorylation, inhibitors of NEC phosphorylation appear attractive for use in novel antiviral targeting strategies [26,59,103,104]. Especially when considering the various structural changes predicted or already proven as a prerequisite for multicomponent NEC assembly and down-stream functions, small molecules interfering with conformational switches might be highly attractive for the pharmacological development [10,20,23]. Thus, an improvement in the understanding of the nuclear egress process and detailed knowledge of herpesviral NEC structure–function relationship will most probably create various novel strategies for NEC-specific drug targeting.

## 9. Conclusions

Herpesviral NECs represent unique assemblies of heterodimeric viral protein pairs (core NEC) that extend to multimeric arrangements (hexameric lattices) and recruit a number of associated effector proteins (multicomponents). Although the basic features of NEC functionality, i.e., the regulated nucleocytoplasmic transition of viral capsids, appears to be a strictly conserved, common concept of all herpesviral core NECs, some specific differences have been identified at the levels of primary sequences and binding properties, when comparing the NECs of individual herpesvirus species or subfamilies. Crystal structures point to a conserved structural basis of the core NECs, whereas the conservation on the basis of primary sequences is limited, i.e., showing a stepwise graduation for NECs of closely or distantly related virus family members, and biochemical properties as well as functional protein interaction behaviors can vary to some extent. At present, the picture emerging from the entity of data published for α-, β- and γ-herpesviral NECs points to a majority of shared properties, but also to several detailed aspects that are distinct between herpesviruses, so that both conclusions appear justified: NECs, a common theme and subtle differences at the same time.

For these reasons, more information is still needed to understand herpesviral NEC functionality in detail, and to possibly utilize this understanding for translational research aimed at pharmacological intervention. The functional importance of the NEC for the efficiency of viral replication has been shown at several levels, strongly indicating that herpesviral NECs represent promising targets for antiviral strategies. This may include both, the formation of core NECs as well as the subsequent assemblies of multicomponent NECs with all associated effector functions. As both types of targets involve virus-specific elements, as well as structures that are conserved among a range of herpesviruses, it is conceivable that NEC inhibitors could prove useful as virus-specific as well as pan-antiherpesviral agents, depending on whether they address a virus-specific or a conserved target within the NECs. Therefore, it can be expected that herpesviral NECs will continue to evolve as attractive and multifaceted objects of antiviral research and drug development, and that a growing understanding of the NECs as central herpesviral control points will in turn enable novel and so far unexploited antiviral strategies.

## Figures and Tables

**Table 1:** Contribution of amino acid side chains and stereochemistry of the HCMV pUL53 hook peptide to its interaction with pUL50.

Alanine Scan		D-amino Acid Scan	
Position	IC_50_ [µM] * ± SD	Position	IC_50_ [µM] * ± SD
WT	0.11 ± 0.04	WT	0.05 ± 0.02
L59	0.89 ± 0.12	L59	0.09 ± 0.01
T60	0.14 ± 0.02	T60	0.20 ± 0.06
L61	0.47 ± 0.01	L61	0.17 ± 0.03
H62	0.13 ± 0.02	H62	0.07 ± 0.01
D63	0.45 ± 0.01	D63	0.54 ± 0.06
L64	>10	L64	1.27 ± 0.31
H65	0.42 ± 0.01	H65	0.11 ± 0.01
D66	0.12 ± 0.001	D66	0.09 ± 0.01
I67	1.30 ± 0.01	I67	2.60 ± 0.06
F68	>10	F68	0.11 ± 0.03
R69	0.15 ± 0.01	R69	0.56 ± 0.03
E70	0.11 ± 0.01	E70	0.08 ± 0.004
H71	0.16 ± 0.03	H71	6.51 ± 0.10
P72	0.32 ± 0.02	P72	4.00 ± 0.22
E73	0.22 ±0.002	E73	0.14 ± 0.02
L74	1.87 ± 0.07	L74	0.75 ± 0.05
E75	>10	E75	>10
L76	0.1 ± 0.003	L76	0.11 ± 0.01
K77	0.37 ± 0.05	K77	3.80 ± 1.12
Y78	>10	Y78	>10
L79	>10	L79	0.94 ± 0.05
N80	0.12 ± 0.01	N80	0.20 ±0.007
M81	1.31 ± 0.13	M81	2.37 ± 0.13
M82	>10	M82	>10
K83	0.22 ± 0.001	K83	0.10 ± 0.02
M84	0.22 ± 0.06	M84	4.61 ± 0.12
A85	0.11 ± 0.04	A85	>10
I86	0.53 ± 0.16	I86	0.86 ± 0.08
T87	0.16 ± 0.05	T87	0.11 ± 0.01

* Inhibition of pUL50–pUL53 interaction; WT, unaltered wild-type sequence.

**Table 2:** Autologous and nonautologous patterns of core NEC protein nuclear rim colocalization: selected examples derived from α-, β- and γ-*Herpesvirinae*. Various combinations of NEC interactions were analyzed on the basis of pairwise transient cotransfection of NEC-encoding expression plasmids, i.e., α-herpesviral pUL34/Orf24, pUL31/Orf27 (HSV-1/VZV); β-herpesviral pUL50/pM50, pUL53/pM53 (HCMV/MCMV); γ-herpesviral BFRF1/Orf67, BFLF2/Orf69 (EBV/ KSHV). Confocal microscopic evaluation of rim-like nuclear envelope colocalization of autologous/ nonautologous core NEC pairs, transiently coexpressed in HeLa cells. Localization phenotypes: -, colocalization in < 5% of cells; ±, 5%-25%; +, 25.1%-75%; ++, >75%.

			Hook Proteins					
			alpha		beta		gamma	
			HSV-1 pUL31	VZV Orf27	HCMV pUL53	MCMV pM53	EBV BFLF2	KSHV Orf69
**Groove Proteins**	alpha	HSV-1 pUL34	++	+	-	-	-	-
		VZV Orf24	+	++	-	-	-	-
	beta	HCMV pUL50	-	-	++	++	-	-
		MCMV pM50	-	-	+	++	-	-
	gamma	EBV BFRF1	-	-	-	-	++	+
		KSHV Orf67	-	-	-	-	+	++

## Supplementary Materials

The following are available online at https://www.mdpi.com/1999-4915/12/6/683/s1. Figure S1. Video depiction of herpesviral 3D core NEC crystal structures: (a) HSV-1, (b) PRV, (c) HCMV and (d) EBV. Figure S2. Primary data of confocal imaging analysis comparing autologous versus nonautologous α-/β-/γ-herpesviral core NEC interactions. Table S1. Sequences of HCMV pUL53 and EBV BFLF2 hook peptides.
