# Supplementary material for: Nuclear Egress Complexes of HCMV and Other Herpesviruses: Solving the Puzzle of Sequence Coevolution, Conserved Structures and Subfamily-Spanning Binding Properties
Source: Viruses. 2020 Jun 24;12(6):683. doi: 10.3390/v12060683 (PMC7354485; doi:10.3390/v12060683)
Supplement: Supplementary file 1 [file viruses-12-00683-s001.zip › 3-viruses-833581-suppl/Supplementary Materials_Marschall et al_Revised.pdf]

viruses

Supplementary Materials

Review

Nuclear egress complexes of HCMV and other herpesviruses: solving the puzzle of sequence coevolution, conserved structures and subfamily-spanning binding properties

Manfred Marschall [1*], Sigrun Häge [1,&], Marcus Conrad [2,&], Sewar Alkhashrom [3,&], Jintawee Kicuntod [1], Johannes Schweininger [4], Mark Kriegel [4], Josephine Lösing [1], Julia Tillmanns [1], Frank Neipel [1], Jutta Eichler [3], Yves A. Muller [4], Heinrich Sticht [2]

1 Institute for Clinical and Molecular Virology, Friedrich-Alexander University of Erlangen-Nürnberg, Medical Center, Erlangen, Germany; manfred.marschall@fau.de.
2 Division of Bioinformatics, Institute of Biochemistry, Friedrich-Alexander University of Erlangen-Nürnberg, 91054 Erlangen, Germany; heinrich.sticht@fau.de.
3 Department of Chemistry and Pharmacy, Division of Medicinal Chemistry, Friedrich-Alexander University of Erlangen-Nürnberg, Erlangen, Germany; jutta.eichler@fau.de.
4 Department of Biology, Division of Biotechnology, Friedrich-Alexander University of Erlangen-Nürnberg (FAU), Erlangen, Germany; yves.muller@fau.de.
& These authors contributed equally to the study.
* Correspondence: manfred.marschall@fau.de; Tel.: +49 9131 85-26089.

Table S1. Sequences of HCMV pUL53 and EBV BFLF2 hook peptides.

Peptide	**Sequence** (position numbers are based on pUL53 and BFLF2, respectively)
pUL53 hook peptide	Ac[a]-[59]LTLHDLHDIFREHPELELKYLNMMKMAIT[87]-Aoa[b]-Lys(Fluo[c])-NH_2
BFLF2 hook peptide	Ac-[78]DRSHFSLRDFFRGISANFELGKDFLREMNTPIH[110]-Aoa-Lys(Fluo)-NH_2

[a] Ac, acetyl; [b] Aoa, 8-amino-3,6-dioxaoctanoic acid; [c] Fluo, fluorescein

Figure S1. Video depiction of herpesviral 3D core NEC crystal structures:
(a) HSV-1, (b) PRV, (c) HCMV and (d) EBV.

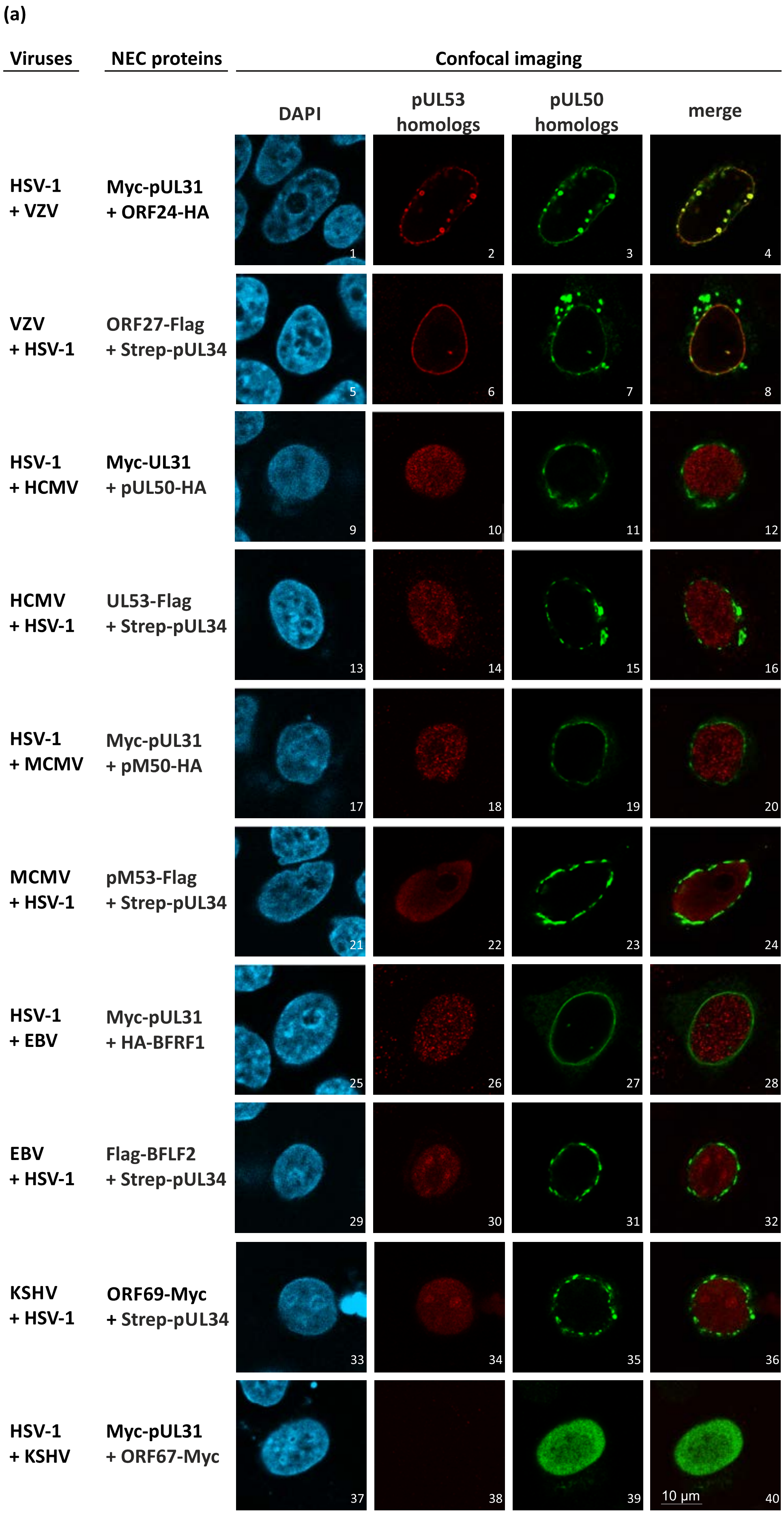
(a)
Viruses
NEC proteins
Confocal imaging
DAPI
pUL53 homologs
pUL50 homologs
merge
HSV-1 + VZV
Myc-pUL31 + ORF24-HA
VZV + HSV-1
ORF27-Flag + Strep-pUL34
HSV-1 + HCMV
Myc-UL31 + pUL50-HA
HCMV + HSV-1
UL53-Flag + Strep-pUL34
HSV-1 + MCMV
Myc-pUL31 + pM50-HA
MCMV + HSV-1
pM53-Flag + Strep-pUL34
HSV-1 + EBV
Myc-pUL31 + HA-BFRF1
EBV + HSV-1
Flag-BFLF2 + Strep-pUL34
KSHV + HSV-1
ORF69-Myc + Strep-pUL34
HSV-1 + KSHV
Myc-pUL31 + ORF67-Myc
10 µm

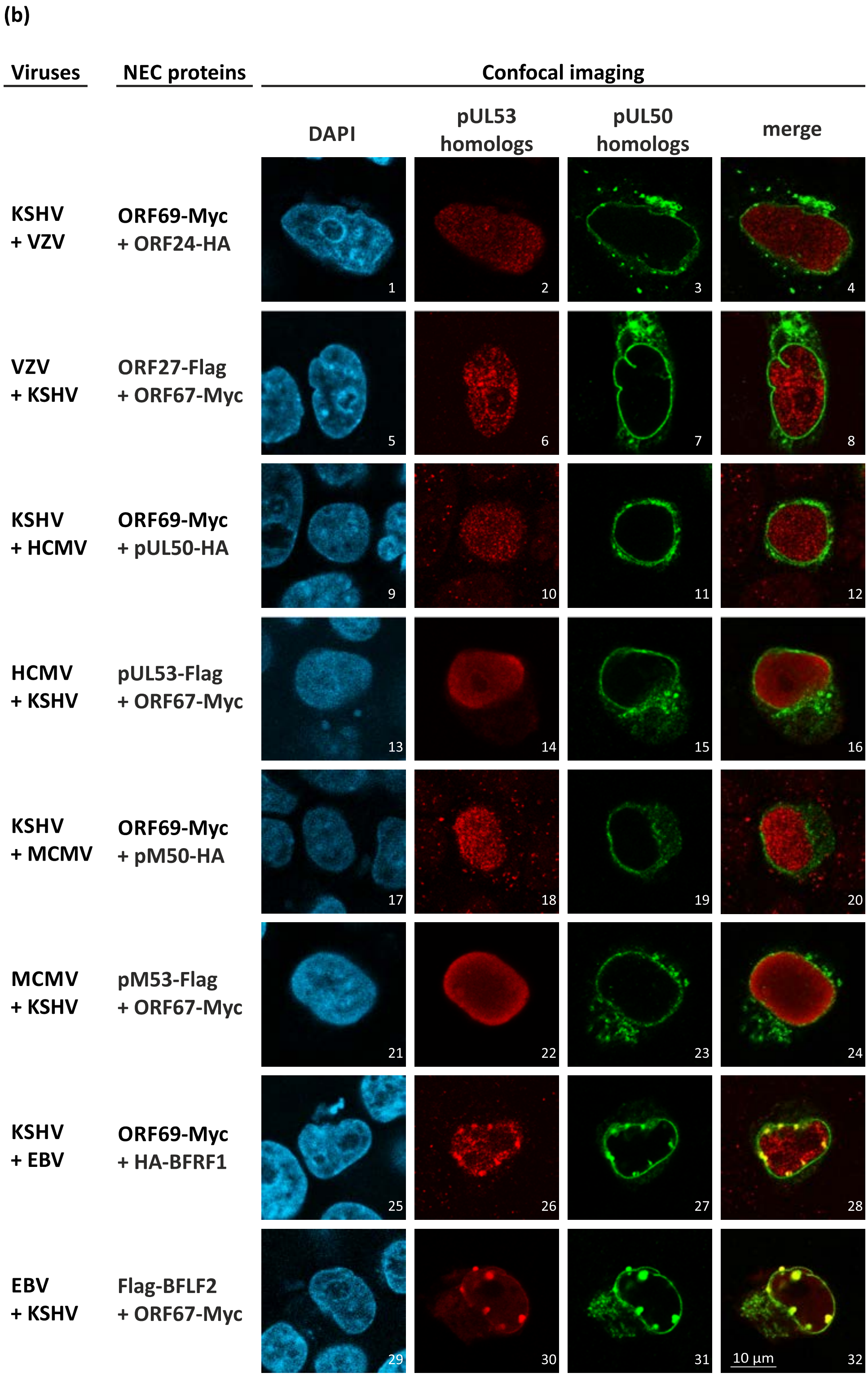

Figure S2. Primary data of confocal imaging analysis comparing autologous versus nonautologous α-/β-/γ-herpesviral core NEC interactions. HeLa cells were transiently transfected with constructs coding for tagged versions of the NEC proteins indicated. Cells were fixed and used for indirect immunostaining with tag-specific primary and fluorescence-labeled secondary antibodies. **(a)** Combinations between HSV-1-specific and various herpesviral NEC proteins. **(b)** Combinations between KSHV-specific and various herpesviral NEC proteins. Scale bar 10 µm.

Materials and Methods

The detailed mechanistic and structural analyses of herpesviral nuclear egress proteins, which have been performed by a great number of independent research groups, are reviewed in Marschall et al. [8]. Materials and Methods used for α-, β- and γ-herpesviruses are specifically referenced therein. For the sequence alignments, the crystal structures of the three subfamily prototypical NEC complexes from HSV-1 (PDB entry code: 4ZXS), HCMV (6T3X), and EBV (6T3Z) were structurally superimposed first and a structure-based sequence alignment deduced. Subsequently, subfamily-specific multiple sequence alignments were calculated and merged with the structure-based sequence alignment. Concerning the generation of primary data sets included in this review article, the Materials and Methods used were published previously [9-12,21-23,25-28]. Peptide synthesis methods [29], as well as the competitive pUL50-pUL53 binding assay to assess the inhibitory activity of the alanine and D-amino acid scan pUL53 hook peptides were previously described [22]. For the fluorescence polarization assay, C-terminally fluoresceinylated pUL53 and BFLF2 hook peptides (25 nM) were incubated with bacterially produced, recombinant pUL50 and BFRF1, respectively, at two-fold serial dilutions, starting at 10 µM (BFRF1) and 20 µM (pUL50), respectively. Fluorescence polarization was measured at 485 nm (excitation) and 535 nm (emission).
